# *SimCP*: A Simulation Platform to Predict Gait Performance Following Orthopedic Intervention in Children With Cerebral Palsy

**DOI:** 10.3389/fnbot.2019.00054

**Published:** 2019-07-17

**Authors:** Lorenzo Pitto, Hans Kainz, Antoine Falisse, Mariska Wesseling, Sam Van Rossom, Hoa Hoang, Eirini Papageorgiou, Ann Hallemans, Kaat Desloovere, Guy Molenaers, Anja Van Campenhout, Friedl De Groote, Ilse Jonkers

**Affiliations:** ^1^Department of Movement Sciences, KU Leuven, Leuven, Belgium; ^2^Department of Rehabilitation Sciences, Doctoral School of Biomedical Sciences, KU Leuven, Leuven, Belgium; ^3^Clinical Motion Analysis Laboratory, University Hospitals Leuven, Leuven, Belgium; ^4^Department of Rehabilitation Sciences and Physiotherapy, University of Antwerp, Antwerp, Belgium; ^5^Department of Orthopedics, University Hospitals Leuven, Leuven, Belgium; ^6^Department of Development and Regeneration, KU Leuven, Leuven, Belgium

**Keywords:** cerebral palsy, muscle synergies, single event multilevel surgery, orthopedic interventions, capability gap, subject specific model

## Abstract

Gait deficits in cerebral palsy (CP) are often treated with a single-event multi-level surgery (SEMLS). Selecting the treatment options (combination of bony and soft tissue corrections) for a specific patient is a complex endeavor and very often treatment outcome is not satisfying. A deterioration in 22.8% of the parameters describing gait performance has been reported and there is need for additional surgery in 11% of the patients. Computational simulations based on musculoskeletal models that allow clinicians to test the effects of different treatment options before surgery have the potential to drastically improve treatment outcome. However, to date, no such simulation and modeling method is available. Two important challenges are the development of methods to include patient-specific neuromechanical impairments into the models and to simulate the effect of different surgical procedures on post-operative gait performance. Therefore, we developed the SimCP framework that allows the evaluation of the effect of different simulated surgeries on gait performance of a specific patient and includes a graphical user interface (GUI) that enables performing virtual surgery on the models. We demonstrated the potential of our framework for two case studies. Models reflecting the patient-specific musculoskeletal geometry and muscle properties are generated based solely on data collected before the treatment. The patient's motor control is described based on muscle synergies derived from pre-operative EMG. The GUI is then used to modify the musculoskeletal properties according to the surgical plan. Since SEMLS does not affect motor control, the same motor control model is used to define gait performance pre- and post-operative. We use the capability gap (CG), i.e., the difference between the joint moments needed to perform healthy walking and the joint moments the personalized model can generate, to quantify gait performance. In both cases, the CG was smaller post- then pre-operative and this was in accordance with the measured change in gait kinematics after treatment.

## Introduction

Cerebral Palsy (CP) is the most common cause of motor deficiency in young children with a prevalence of 2–3 cases per 1,000 live births (Fairhurst, 2012; Colver et al., 2014; Graham et al., 2016). Due to lesions in the developing brain, children with CP display motor disabilities that vary greatly in presentation and severity. While CP is not a progressive disease, with time, secondary symptoms might arise, such as bony deformities and muscle contractures. Alongside increasing pain and fatigue, these symptoms can pose severe limitations to the quality of life and independence of the patients (Hanna et al., 2009; Opheim et al., 2009). Nowadays, several orthopedic treatments, often in combination with physical therapy and orthoses, are available and aim at improving the functionality and therefore quality of life of these patients (Fairhurst, 2012; Narayanan, 2012; Fitoussi and Bachy, 2015; Strobl et al., 2015; Nieuwenhuys et al., 2016).

For ambulatory patients, orthopedic treatments usually aim at improving walking speed and stability, at reducing the need of walking aids and at mitigating or preventing fatigue and pain (Narayanan, 2012). The selection of the most appropriate surgical treatment is a complex endeavor that nowadays is mainly based on the clinical assessment of the patient, integrated 3D gait analysis and medical imaging (Molenaers et al., 2001; Strobl et al., 2015). The outcome, however, is not always as desired and studies reported a deterioration in 22.8% of the parameters used to describe gait performance after surgery (Filho et al., 2008). In 11% of the cases, additional surgeries are needed to improve the functional outcome, although, this can be as high as 32% when no gait analysis is used to support the decision-making process (Wren et al., 2009).

It is therefore of the utmost importance to identify the parameters that determine the success of an orthopedic intervention (Hersh et al., 2002; Arnold et al., 2006a; Niiler et al., 2007; Fox et al., 2009; Reinbolt et al., 2009; Hicks et al., 2011; Schwartz et al., 2013, 2016; Mansouri et al., 2016; Galarraga et al., 2017). This would allow making pre-operative predictions in order to guide the decision-making process toward the most effective treatments in terms of functional outcome. Several studies applying statistical approaches and more recently machine learning methods to explore these relationships (Hersh et al., 2002; Reinbolt et al., 2009; Hicks et al., 2011; Schwartz et al., 2013, 2016) have been quite successful in predicting the improvement or non-improvement of a few selected outcome indicators when dealing with selected surgeries. However, existing methods do not produce a comprehensive outcome prediction and do not account for combinations of different surgeries. Notably, Galarraga et al. (2017) developed a method based on dimension reduction and multiple linear regression to predict lower limb kinematics for a large number of surgical procedures. All these methods, however, have the drawback that they are black box methods and therefore do not allow investigating the mechanisms relating outcomes in motor function to the specific interventions (Halilaj et al., 2018). On the other hand, methods relying on musculoskeletal models and computational simulations are often suggested to have the potential to identify the causal relation between individual impairments, their interactions and the treatment outcome (Morrison et al., 2018).

To introduce simulation-based decision-supporting tools into clinical practice, a few obstacles have yet to be overcome. One of the major obstacles in this respect is the need for a representative translation of the neuromusculoskeletal dysfunctions of the patients (i.e., the altered musculoskeletal geometry, musculoskeletal parameters, and altered neural control) into the musculoskeletal models. The need to account for the musculoskeletal deformities of the individual CP patient and the bony deformities in particular, dictates the use of *subject-specific musculoskeletal models* when generating dynamic simulations of CP gait. In this respect, the added value of magnetic resonance imaging (MRI) based models has been extensively demonstrated (Scheys et al., 2011a,b; Bosmans et al., 2016).

The altered muscle parameters (i.e., muscle contracture and weakness) in patients with CP compared to a healthy population (Theis et al., 2016; Kruse et al., 2017; Kalkman et al., 2018) invalidates the use of scaled generic parameters. Appropriate parameter tuning capturing the patient-specific muscle properties is therefore needed. Several methods have been proposed for tuning and scaling musculoskeletal parameters (Van Campen et al., 2014; Modenese et al., 2016; Falisse et al., 2017). Nevertheless, most of these methods require an extensive amount of data collected based on a specific method (e.g., instrumented dynamometry) that is typically not available in the common clinical practice and might be difficult to apply in the case of neuromotor deficits.

The altered motor control of patients with CP is reflected in the use of aberrant coordination patterns of the muscles during gait compared to a healthy population (Steele et al., 2015). The concept of muscle synergies is an elegant way to summarize these coordination patterns and by comparing them between CP and typically developing (TD) children, altered motor control aspects have already been identified in terms of number of independent components and stride-by-stride variability (Steele et al., 2015; Kim et al., 2018). In addition, muscle synergies have already been used in the control of musculoskeletal models during dynamic simulations (Allen and Neptune, 2012; Sartori et al., 2013; Meyer et al., 2016). In healthy subjects, the muscle activations generating the observed muscle synergies are very similar to those generated when muscles are recruited independently according to an optimality criterion (De Groote et al., 2014). However, patients with CP exhibit different sets of muscle synergies with respect to a healthy population (Steele et al., 2015), thus highlighting the importance of including a subject-specific motor control model into the framework (Meyer et al., 2016; Sartori et al., 2017).

Literature results (Patikas et al., 2007) and a pilot study from our research group (Pitto et al., 2018), suggest that the same motor control model can be used to describe both the pre- and post-operative patient's condition. Therefore, the pre-operative synergies may also be used for the simulations of the post-operative condition, as their composition remains mostly unchanged after a specific orthopedic treatment. The advantage of this approach is that it relies entirely on pre-operative data, thus making it suitable for the pre-operative decision-making process.

Apart from describing the patient-specific features in the modeling framework, also the specific therapeutic interventions (and multi-level surgeries in particular) need to be accounted for into the musculoskeletal model. Whereas the effect of muscle-tendon lengthening and muscle transfer on the moment-generating capacity, lengths and velocities of the muscles have been described (Delp and Zajac, 1992; Arnold et al., 2006b), only few studies attempted a forward simulation aiming to predict the post-intervention outcome. For instance, two studies using musculoskeletal modeling and forward dynamics simulations (Fox et al., 2009; Mansouri et al., 2016) investigated the effect of a rectus femoris transfer surgery on the recovery of balance after a perturbation and on knee flexion in stiff knee gait in children with CP. While these studies represent a step forward in this direction, their scope remains quite restricted, accounting for only one kind of intervention and analyzing the effect on a single parameter.

Clinical use of simulation-based decision-supporting tools requires the definition of comprehensive parameters that relate to the functional improvement of the patient and therefore can be used as outcome measures to evaluate the effect of different interventions. In the field of assistive exoskeletons (Afschrift et al., 2014), the concept of the capability gap (CG) was introduced to represent the amount of support the exoskeleton had to provide in order to allow the patient to perform a given task. This concept can be translated to the estimation of the motor performance of the patient, before and after the simulated interventions, as a measure of “difficulty” in performing a desired motion, i.e., gait pattern of a TD child. By integrating the patient-specific impaired motor control, abnormal muscle properties, and/or altered musculoskeletal geometry, the changes in the CG after a simulated orthopedic treatment inform the clinician on how a specific intervention improves the ability of the child to adopt a TD gait pattern.

**Figure 1:**
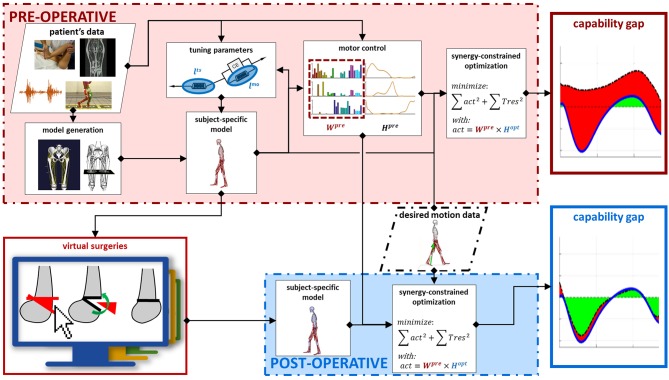
Framework description. Data collected on the patient in the pre-operative phase are used as input to generate a pre-operative model with personalized musculoskeletal geometry and muscle parameters. Next, the motor control of the patient is modeled, using synergy decomposition analysis, based on pre-operative EMG and gait analysis data. Performing virtual surgeries on the pre-operative model generates a post-operative model. This takes into account the changes in the musculoskeletal parameters induced by the performed surgery. The motor control, on the other hand, is not affected by the surgery and the same motor control model computed in the pre-operative condition is used also for the post-operative condition. The gait performance of the patient can be computed in both the pre- and simulated post-operative conditions. In both cases, a synergy constrained optimization tries to match the joint moments relative to a desired motion while taking into account the constraints imposed by the model and by the motor control. The differences between the moments generated by the model and the desired moments are the capability gap, defining the gait performance.

Within the SimCP project, a comprehensive simulation-based framework was developed to evaluate the functional effect of a therapeutic/surgical intervention in a specific patient with CP, thereby assisting the most appropriate treatment selection (Figure 1). This framework relies on the creation of a personalized neuro-musculoskeletal model of the patient. In this model, the musculoskeletal geometry is obtained from imaging data. The framework then provides the tools to personalize the muscle parameters according to information collected during gait analysis and clinical examination. Furthermore, the motor control is personalized using EMG data collected during the treatment-planning phase. Thereafter, a Graphical User Interface (GUI) allows clinicians to simulate combinations of different multi-level surgical procedures. Finally, the functional performance (i.e., walking ability) of the patient can be quantified for different simulated post-operative conditions by evaluating the change in the predicted capability gap with respect to the pre-operative condition. These operations rely only on experimental data collected pre-operative. In this manner, it is feasible to compare the effectiveness of a set of candidate treatments in improving the gait performance of the patient, thus supporting the clinical decision-making process and optimizing individual treatment outcome.

Throughout this article, the different building blocks composing the framework are described and two representative cases studies are introduced to assess the methods and elucidate the several steps.

## Methods

The goal of the SimCP framework is to predict gait performance following different candidate orthopedic treatments solely based on data collected before the treatment and the surgical plan. Pre-operative data includes gait analysis, clinical examination (e.g., documenting joint range of motion) and medical images. To this aim, the framework contains several building blocks (Figure 1). First, a personalized musculoskeletal model capturing the patient's musculoskeletal geometry (section Musculoskeletal Geometry) and muscle properties (section Muscle Parameters) is generated. Then, a description of the patient's motor control is added to this model (section Motor Control). Next, the orthopedic surgeries are simulated (section Surgery Simulation). Finally, the simulated post-operative gait performance is computed (section Capability Gap and Muscle Report). The framework also offers the possibility to investigate the contributions of the different impairments to gait performance (section Alternative Analyses). We illustrate the salient features of the SimCP framework using data from two representative patients (section Case Studies).

### Data Collection

In order to provide the information needed for the complete personalization of the models, inputs from several sources are required. First, imaging data, such as CT scans and MRI-images, allow defining the musculoskeletal geometry. Second, three-dimensional gait analysis data, including marker trajectories and ground reaction forces as well as EMG signals from the most important muscles in the lower limb, are needed for the personalization of the muscle parameters and definition of the motor control model. Third, clinical examination reporting the passive range of motion of the patient contains useful information to refine the personalization of the muscle parameters.

### Musculoskeletal Geometry

The musculoskeletal models are based on a generic SIMM (Motion Analysis Corp., Santa Rosa, CA) model and are composed of 14 bodies and 21 degrees of freedom that are actuated by 86 muscles. The musculoskeletal geometry of this generic model is adapted to reflect the patient's musculoskeletal geometry. The model is then further personalized by tuning the muscle-tendon parameters (section Muscle Parameters) and by adding a model of the patient's motor control (section Motor Control).

Musculoskeletal geometry is derived from MRI-images. The workflow to create the MRI-based models has been published previously (Scheys et al., 2008, 2011b). In short, bones of the lower limbs and pelvis are segmented using Mimics (Materialize, Leuven, Belgium). Afterwards, anatomical reference frames, joint axes and muscle origin and insertion points are defined and a patient-specific musculoskeletal OpenSim model is created using MuscleSegmenter (Leuven, Belgium) and customized Matlab (The Mathworks, Natick, MA) scripts. The models with personalized musculoskeletal geometry are then imported into the SimCP framework.

Performing virtual surgery alters the musculoskeletal geometry and hence generates a new model, which is linked to the *pre-operative* model and appears in the *post-operative* model list. In this manner, each *pre-operative* model can be linked to multiple *post-operative* models, allowing for the exploration of different treatment options (see also section Surgery Simulation).

### Muscle Parameters

Using preoperative data, we tune the two parameters of the Hill-type muscle model that have the largest influence on the simulated muscle force: muscle optimal fiber length and tendon slack length (*l*^*mo*^and *l*^*ts*^) (De Groote et al., 2010). Maximum isometric muscle forces are scaled based on the patient's body weight (Van Der Krogt et al., 2016; Kainz et al., 2018). Pennation angles are taken from the gait2392 model in OpenSim as they have a limited effect on simulated forces (Zajac, 1989).

Parameters are tuned using only the information from the pre-operative walking trials (marker trajectories, ground reaction forces and EMG) and, optionally, the clinical examination of the passive joint range of motion (Table 1). The underlying assumption of the procedure is that during gait the muscles generate the inverse dynamic moments with activations that are consistent with the measured EMGs and operate around their optimal fiber length. In other words, given the joint excursions during gait, muscles are not extremely short, as this would limit their force production given the muscle's force length relationship, nor too stretched, as this would induce excessive passive forces. In addition, it is assumed that at least part of the resistance encountered during the clinical examination at the extremes of the range of motion, is attributable to muscle passive force, i.e., muscles being at a length well above their optimal length.

**Table 1:** Subjects demographics and data from clinical examination.

	**Patient 1**		**Patient 2**		**TD (15 subjects)**	
Age	12–16 years		12–16 years		9.86 (SD 2.98) years	
Weight	33.1 kg		49.1 kg		34.61 (SD 13.33) Kg	
Height	143 cm		171 cm		139 (SD 166) cm	
Time between observation	407 days		304 days			
		**Left**	**Right**	**Left**	**Right**	**Reference values (Moon et al.**, **2017****)**
**Passive range of motion (degrees)**	Hip flexion	145 ~ **/**	140 ~ **/**	105 ~ **/**	110 ~ **/**	126.8 (SD 7.6)
	Hip extension	−10 ~ **/**	−10 ~ **/**	/ ~ **/**	/ ~ **/**	
	Hip abduction (Knee 0°)	25 ~ **40**	25 ~ **40**	20 ~ **15**	10 ~ 2**0**	47.6 (SD 6.2)
	Hip abduction (Knee 90°)	45 ~ **35**	45 ~ **35**	35 ~ **25**	30 ~ 3**0**	55.6 (SD)
	Hip adduction	0 ~ **/**	0 ~ **/**	0 ~ **/**	0 ~ **/**	
	Hip int rotation (prone)	60 ~ **40**	70 ~ **60**	45 ~ **25**	65 ~ **25**	40.1 (SD 11.1)
	Hip ext rotation (prone)	25 ~ **20**	25 ~ **20**	20 ~ **5**	20 ~ **5**	40.1 (SD 8.5)
	Hip int rotation (supine)	25 ~ **25**	30 ~ **40**	30 ~ **20**	45 ~ **25**	
	Hip ext rotation (supine)	55 ~ **50**	50 ~ **40**	45 ~ **20**	30 ~ **10**	
	Knee flexion	120 ~ **110**	120 ~ **105**	120 ~ **/**	120 ~ **/**	136.5 (SD 5.5)
	Knee extension	−20 ~ **0**	−15 ~ **10**	10 ~ **5**	−25 ~ **0**	1.0 (SD 1.8)
	Knee spontaneous position	−30 ~ **5**	−25 ~ **5**	/ ~**−10**	/ ~ –**10**	
	Popliteal angle unilateral	−70 ~ **135**	−65 ~ **142**	−75 ~**−70**	−85 ~**−70**	33.8 (SD 10.3)
	Popliteal angle bilateral	−65 ~ **135**	−60 ~ **142**	−70 ~**−70**	−75 ~**−70**	24.3 (SD 9.1)
	Ankle dorsiflexion (Knee 90°)	20 ~ **30**	25 ~ **30**	20 ~ **30**	−10 ~ **10**	19.6 (SD 4.5)
	Ankle dorsiflexion (Knee 0°)	15 ~ **20**	15 ~ **20**	10 ~ **15**	−20 ~ **0**	11.3 (SD 4.7)
	Ankle plantarflexion	35 ~ **discr**	35 ~ **discr**	10 ~ **norm**	20 ~ **norm**	49.4 (SD 9.2)
	Ankle inversion	40 ~ **norm**	45 ~ **norm**	50 ~ **norm**	60 ~ **norm**	
	Ankle eversion	10 ~ **norm**	10 ~ **norm**	10 ~ **norm**	10 ~ **norm**	
**Spasticity**	Hip flexion Mas	2 ~ **0**	2 ~ **0**	1.5 ~ **1**	2 ~ **1**	
	Hip adduction (Knee 0°) mas	1.5 ~ **0**	1.5 ~ **0**	1.5 ~ **1**	2 ~ **1**	
	Hip adduction (Knee 90°) mas	0 ~ **0**	0 ~ **0**	1.5 ~ **1**	2 ~ **1**	
	Hamstrings mas	1.5 ~ **0**	1 ~ **0**	2 ~ **2**	1.5 ~ **1.5**	
	Hamstrings tard	−70 ~ **/**	/ ~ **/**	−85 ~**−90**	−90 ~**−75**	
	DuncanElly mas	1.5 ~ **0**	1.5 ~ **0**	1.5 ~ **1.5**	2 ~ **1.5**	
	DuncanElly tard	2 ~ **0**	2 ~ **0**	2 ~ **2**	2 ~ **2**	
	Soleus mas	0 ~ **/**	0 ~ **/**	1.5 ~ **1.5**	1.5 ~ **2**	
	Soleus tard	/ ~ **/**	/ ~ **/**	10 ~ **10**	−15 ~ **0**	
	Gastrocnemius mas	1.5 ~ **/**	1.5 ~ **/**	3 ~ **1.5**	3 ~ **2**	
	Gastrocnemius tard	0 ~ **/**	5 ~ **/**	−20 ~**−10**	−25 ~**−10**	
	Tibialis post mas	0 ~ **0**	0 ~ **0**	0 ~ **0**	2 ~ **1.5**	
	Clonus	0 ~ **/**	0 ~ **/**	2 ~ **2**	3 ~ **2**	
	Plantarflexors (Knee 90°) mas	/ ~ **1**	/ ~ **1**	/ ~ **/**	/ ~ **/**	
	Plantarflexors (Knee 90°) tar	/ ~ **10**	/ ~ **10**	/ ~ **/**	/ ~ **/**	
	Plantarflexors (Knee 0°) mas	/ ~ **0**	/ ~ **0**	/ ~ **/**	/ ~ **/**	
**Selectivity**	Hip flexion	2 ~ **2**	2 ~ **2**	2 ~ **2**	2 ~ **2**	
	Hip extension	1.5 ~ **1**	1.5 ~ **1**	2 ~ **2**	1.5 ~ **2**	
	Hip abduction	1.5 ~ **2**	1.5 ~ **1.5**	2 ~ **2**	2 ~ **1.5**	
	Hip adduction	2 ~ **2**	2 ~ **2**	2 ~ **2**	2 ~ **2**	
	Knee flexion	1.5 ~ **2**	1.5 ~ **2**	1.5 ~ **1.5**	1.5 ~ **1.5**	
	Knee extension	1 ~ **2**	1.5 ~ **2**	1.5 ~ **1.5**	1.5 ~ **1.5**	
	Ankle dorsiflexion (Knee 90°)	1.5 ~ **1.5**	1.5 ~ **1.5**	2 ~ **1.5**	1.5 ~ **1.5**	
	Ankle dorsiflexion (Knee 0°)	1.5 ~ **2**	1.5 ~ **2**	1.5 ~ **1.5**	1.5 ~ **1.5**	
	Ankle plantarflexion	1.5 ~ **2**	1.5 ~ **2**	1.5 ~ **2**	1.5 ~ **1.5**	
	Ankle inversion	1.5 ~ **1.5**	1.5 ~ **2**	2 ~ **2**	1.5 ~ **1.5**	
	Ankle eversion	2 ~ **1.5**	1.5 ~ **1.5**	2 ~ **2**	1.5 ~ **1**	
**Strength**	Hip flexion	4 ~ **4**	4 ~ **4**	5 ~ **4**	5 ~ **4**	
	Hip extension	3 ~ **3+**	3 ~ **3+**	3 ~ **4**	4 ~ **4**	
	Hip abduction	3 ~ **3+**	3 ~ **2**	4 ~ **4**	3 ~ **3**	
	Hip adduction	4 ~ **4**	4 ~ **4**	5 ~ **5**	4 ~ **5**	
	Knee flexion	4 ~ **4**	3 ~ **4**	4 ~ **4**	4 ~ **4**	
	Knee extension	3 ~ **4**	3 ~ **4**	4~ **4**	4 ~ **4**	
	Ankle dorsiflexion (Knee 90°)	4 ~ **4**	4 ~ **4**	4 ~ **4**	3 ~ **4**	
	Ankle dorsiflexion (Knee 0°)	4 ~ **4**	4 ~ **4**	4 ~ **4**	3 ~ **4**	
	Ankle plantarflexion	4 ~ **4**	3 ~ **4**	3 ~ **4**	3 ~ **3**	
	Ankle inversion	4 ~ **3+**	4 ~ **4**	5 ~ **4**	3 ~ **4**	
	Ankle eversion	4 ~ **3+**	4 ~ **3**+	4 ~ **4**	3 ~ **3**	

*Information about the subjects included in the study. Data are collected from the pre- and post-operative clinical reports. For each entry, the first value is relative to the pre-operative condition, the second, in bold, is relative to the post-operative condition. “Mas” stands for manual Ashworth test (a score of 0 indicates no spasticity, a score of 4 maximal spasticity), “Tard” for Tardieu test (measured in degrees, defines the position where the spasticity limits the movement). In “Selectivity” a score of 2 indicates the maximum motor control selectivity. In “Strength” the maximum value is represented by a score of 5. “norm” stands for normal, defining full range of motion; “discr” stands for discrete, defining limited range of motion; “/” replaces values not present in the report. Reference values for TD subjects are obtained from Moon et al. (2017)*.

To perform this tuning, we extended a static optimization problem since this allows us to optimize the fit between the computed activations and joint moments, and, respectively, the EMGs and inverse dynamic joint moments. Static optimization computes muscle activations that produce the inverse dynamic joint moments underlying a measured movement while optimizing a performance criterion (e.g., minimizing sum of activations squared). Here, we allow optimal fiber lengths and tendon slack lengths to change during the optimization while imposing constraints on the allowable muscle lengths that represent the tuning criteria described above. In contrast to the typical static optimization approach that is solved for each time frame separately, here all time frames are coupled to obtain a single set of muscle-tendon parameters. It is important to note that static optimization neglects muscle dynamics by assuming that tendons are rigid but allows accounting for the muscle force-length-velocity relationship (De Groote et al., 2016). A static optimization approach was preferred over a dynamic approach that accounts for muscle dynamics (De Groote et al., 2016) to limit computation times. The problem was then solved using the *fmincon* function in Matlab.

To cope with the scarcity of input data (i.e., data from a limited number of movements and a limited number of EMG signals), we decided to tune the parameters only in a set of major muscles (*M*) (Table 2). In addition, we used a different level of detail when describing the force generated by these major and other muscles. For the major muscles, the force-length relationship, derived from (De Groote et al., 2016), was taken into account (but not the force-velocity relationship). Hence, the generated force has an active component (*f*^*L*^), depending on normalized muscle fiber length ($\tilde{l}$) and muscle activation (*a*), and a passive component, (*f*^*P*^) depending only on fiber length:

(1) Fmi=fm∘[amifmiL(l˜mi)+fmiP(l˜mi)], ∀m ∈M,

Where *f* is the maximum isometric force the muscle can exert and the subscript *i* defines the instant in time. For the remaining muscles (*N*), the generated force is proportional to activation:

(2) fmi=fm∘ami,  ∀m∈N.

Hence, the resulting estimation problem has the following structure. Optimization variables consist of muscle activations and reserve moments (for each joint *j*) during the gait cycle (ami,τjiR), as well as the muscle parameters (^*m*^*l*^*mo*^, ^*m*^*l*^*ts*^). Reserve moments are generated by ideal actuators and are added to the muscle moments to guarantee that the inverse dynamic joint moments can be matched even when the muscles are not sufficiently strong. Since they are not physiological, their use is heavily penalized in the cost function to keep their contribution to a minimum:

(3) CSO=∑i(∑mw1(ami)2+∑jw2(τjiR)2),

Where *w*_1−2_ are weighting coefficients that produced a proportional balance between muscle and residual activations (Hicks et al., 2015) in simple static optimization problems.

**Table 2:** List of muscle subsets.

**Tuned muscles**	**EMG muscles**		**Passive range of motion**	
	**EMG channel**	**Muscle name**	**Measurement**	**Muscles measured**
	Rectus Fems	Rectus Fem	**Hip**	
Glut Max 1				
Glut Max 2				
Glut Max 3	Vast Lat	Vast La	Flexion	Glut Max1, Glut Max2, Glut Max3,
Glut Med 1	Bic Fem	Bic Fem lh		Glut Med1, Glut Med2, Glut Med3
Glut Med 2	Hamstring Med	Semimembr, Semitend	Extension	Iliacus, Psoas
Glut Med 3	Tibialis anterior	Tibialis Ant	Abduction 0^°^	Add Mag 2, Add Mag 3, Add Long
Add Long	Gastrocnemius	Gastroc Med, Gastroc Lat	Int Rot Sup	Glut Med1, Glut Med2, Glut Med3
Add Mag 2	Soleus	Soleus	Int Rot Pro	
Add Mag 3	Gluteus	Glut Med 2	Ext Rot Sup	
Tensor FL			Ext Rot Pro	
Gracilis			**Knee**	
Semimembr			Flexion	Rectus Fem, Vast Int, Vast Med, Vast Lat
Semitend			Rectus Fem	
Bic Fem lh			Extension	Semimembr, Semitend, Gracilis, Bic Fem lh, Bic Fem sh
Bic Fem sh			Popl Ang Uni	
Sartorius			Popl Ang Bi	
Rectus Fem			**Ankle**	
Vast Med			Dorsiflex Kn 0^°^	Soleus, Gastroc Lat, Gastroc Med
Vast Int			Dorsiflex Kn 90^°^	
Vast Lat				
Gastroc Med				
Gastroc Lat				
Soleus				
Iliacus				
Psoas				

“*Tuned muscles” reports the set of muscles whose parameters have been tuned; “EMG muscles” reports which muscles of the model were assigned to which EMG channels; “Passive Range of motion” reports the clinical examination measures included into the framework and which muscles were measured in each position*.

An additional penalty term is included in the cost function to ensure that the computed activations of the subset of muscles (ε) for which EMG was collected (see Table 2) reflect the pattern of the measured data:

(4) C∈=∑i∑m∈∈w3(σmami−εmi)2,

Where ε represents the experimental EMG envelope. The scaling factor σ was introduced as an optimization variable to impose similarity between activations and EMG patterns irrespective of signal amplitude since, in the absence of maximum voluntary contraction tests, the relation between signal amplitude and muscle activation cannot be accurately derived.

The cost function was minimized subject to the following constraints. A first set of constraints describes that the muscles should produce the inverse dynamic joint moments:

(5) ∑m∈Mrjmi Fmi + ∑m∈Nrjmi fmi+τjiR−τjiID=0,

Where τjiID are the desired joint moments from inverse dynamics and rjmi is the moment arm of muscle *m* with respect to joint *j* at time *i*.

A second set of constraints imposes bounds on the muscle fiber lengths during gait and the clinical exam of the range of motion. To ensure that normalized muscle fiber lengths during gait are within 0.4 and 1.5, we constrain the minimal and maximal fiber lengths. In addition, to ensure that muscles operate around their optimal length during gait, we constrain the maximal fiber length to be above and the minimal fiber length to be below optimal fiber length:

(6) 1<maxi(l˜mi)≤1.5,

(7) 0.4≤mini(l˜mi)<1,   ∀m∈M,

Maximal normalized fiber lengths during the clinical exam (${\tilde{l}}^{R}$) should be in the range where passive force is generated:

(8) 1<l˜mR≤1.5,   ∀m∈R,

Where *R* defines the subset of muscles for which the length is computed using information from the clinical passive range of motion examination (Table 2).

To impose that muscles were stretched to a level where they generated considerable passive force during the clinical exam, we added a penalty term to the cost function:

(9) CR=∑m∈Rw4(l˜mR−1.5)2,

Within our formulation, the passive force exerted by a muscle stretched at 1.5 *l*^*mo*^ is around 0.5 *F*. While a significant variation in passive force is present between muscles (Prado et al., 2005) and further variations are induced by CP (Kalkman et al., 2018), we made this simplifying assumption to allow for the selection of different sets of muscles without increasing the complexity of the tuning procedure.

Combining, (Equations 3, 4, and 9) and, the final cost function becomes

(10) $$C^{SO}+C^{\epsilon }+C^{R},$$

Input data τjiID, rjmi, and muscle-tendon lengths lmimt are computed based on the personalized musculoskeletal models using OpenSim's analysis tools, specifically Inverse Kinematics, Inverse Dynamics and Muscle Analysis using the gait data as input. Therefore, muscle moment arms are defined using the generalized force method (Sherman et al., 2014). Muscle fiber lengths (*l*) and normalized fiber length ($\tilde{l}$) during gait were computed according to a Hill-type model assuming a rigid tendon:

(11) lmi=(lmimt−lmts)/cos(α),

(12) l˜mi=lmi/lmmo,  ∀m∈M,

Where α is the pennation angle. Joint positions during the clinical exam to test the passive range of motion are derived from the description of the test and are used to compute the maximum musculotendon lengths, muscle fiber lengths and normalized fiber lengths reached during the test for the subset of muscles *R* (*l*^*mtR*^, *l*^*R*^ and ${\tilde{l}}^{R}$, respectively).

The estimated parameters *l*^*mo*^and *l*^*ts*^ are incorporated into the subject's model and will define the force-length relationships of the muscles included in the set *M* during the following analyses.

### Motor Control

In cerebral palsy, the ability to selectively recruit muscles is reduced. Therefore, we describe impaired motor control by imposing the pathological muscle activation patterns when computing gait performance. Muscle activation patterns are derived from pre-operative EMG data collected during walking using synergy analysis. For the motor control model to be useful in our simulations, it has to account for the activations of all the muscles. However, EMG data are collected from only a small subset of muscles (typically <10) while the musculoskeletal model contains many more muscles (typically more than 40). A multi-step procedure is proposed to derive muscle coordination for all muscles in the model based on EMG data from a limited number of muscles (cfr. Meyer et al., 2016).

First, muscle synergies are derived from the pre-operative EMG data acquired during three gait cycles to define the complexity of the patient's motor control by the number of synergies (*N*^*s*^) using non-negative matrix factorization (NNMF) (Lee and Seung, 1999). The input matrix of EMG signals has dimensions *N*^ϵ^ × *N*^*i*^ where *N*^ϵ^ is the number of muscles and *N*^*i*^ is the number of time instants. The output of the synergy analysis consists of two matrices: a *N*^*s*^ × *N*^*i*^ matrix *H* containing the activation timing profiles of each synergy and a *N*^ϵ^ × *N*^*s*^ matrix *W* containing the weight vectors specifying how much an individual muscle is activated by each synergy. The matrices *W* and *H* are computed such that the product *WH* best approximates the original input matrix for a predefined number of synergies *N*^*s*^. We quantified *N*^*s*^ using a bootstrapping procedure such that the percentage of the original signal explained by the synergies is above a predefined threshold (Cheung et al., 2009). Both the EMG signal and the *H* matrix were consistently resampled 500 times with replacements, using Matlab function *datasample*. The resampled matrices have the same dimension of the original ones and the same time instants of the original matrices can appear more than once in the resampled one. The variability accounted for (VAF) of these resampled signals by the synergies extracted from the original signal is computed. *N*^*s*^ is the lowest number of synergies for which VAF is higher than 90% for at least 95% of the resampled signals. The number of synergies *N*^*s*^ is used in the subsequent steps.

Next, an EMG-informed static optimization analysis is performed on the patient's pre-operative gait data, where the optimization variables are ami,τjiR, and ^*m*^σ. The cost function is obtained by combining (Equations 3 and 4):

(13) $$C^{SO}+C^{\epsilon },$$

The *C*^ϵ^ term enables us to account for the pathological characteristics of muscle activations, such as antagonistic muscle co-contractions, which are very common in children with CP. This cost is minimized subject to constraints describing the equilibrium between inverse dynamic and muscle moments (Equation 5).

Finally, muscle synergies are extracted by performing a new NNMF on the muscle activations computed with static optimization. The number of synergies for this analysis is *N*^*s*^ + 1. The extra synergy is included to take into account muscles for which no surface EMG was collected. For instance, Allen and Neptune (2012) found that a synergy including predominant contributions from the iliacus and psoas muscles is needed to control a 3D model during walking, and EMG from these muscles is typically not acquired. The result is a set of muscle synergies ($W_{\;}^{pre}$and *H*^*pre*^) that define the motor control model of the patient and describe the activations of all the muscles. This motor control model is later used in the computation of the gait performance of the patient (section Capability Gap and Muscle Report).

### Surgery Simulation

We developed a set of virtual surgeries and a GUI that allow to directly manipulate the musculoskeletal models by leveraging the Matlab-OpenSim application programming interfaces. The surgeries implemented in the current version of the GUI are *Extension and Derotation Osteotomy, Derotation Osteotomy, Muscle Transfer, Patella Advancement* and *Botulinum Toxin Injection*. Only the *Muscle Transfer* and *Botulinum Toxin Injections* influence the muscle parameters.

**Figure 2:**
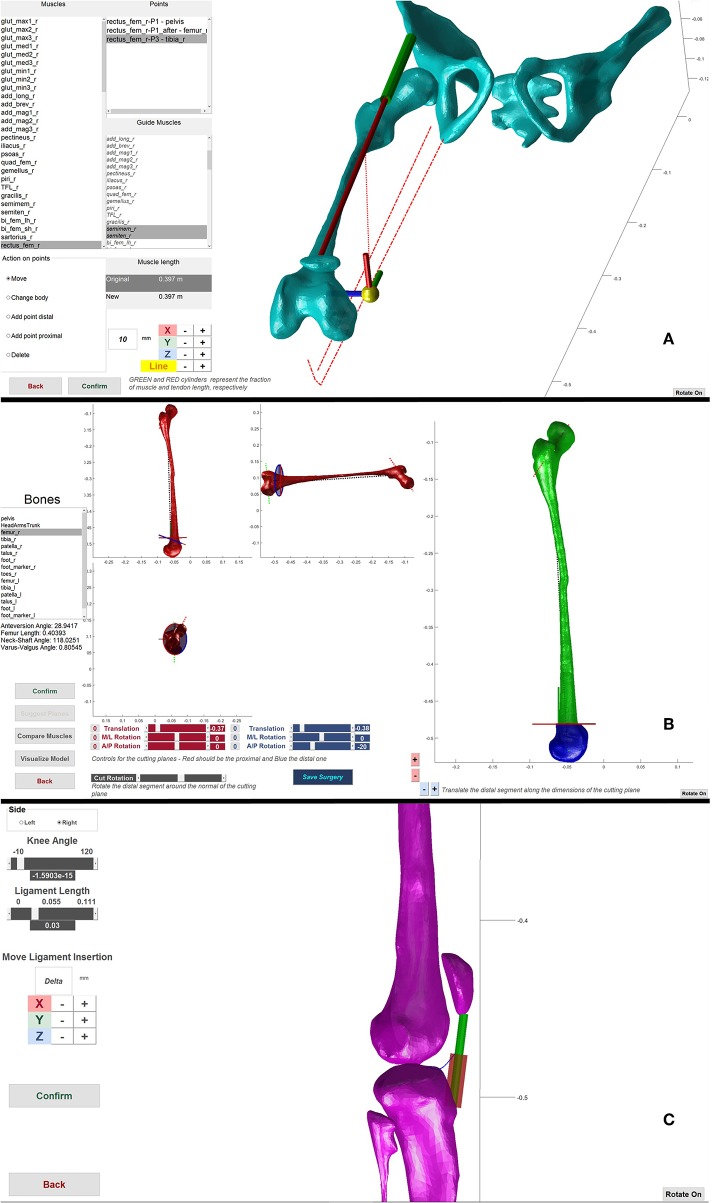
Framework components. **(A)** Muscle transfer surgery. Used to change the path of a muscle. The lines of action of other muscles are visualized as guide to help during the transfer. **(B)** Extension and Derotation osteotomy surgery. Two sets of scrollbars define the pose of the two cutting planes (red and blue in the left side figures) that define the wedge of bone to be removed. After wedge removal, the two segments are brought in contact and it is possible to rotate and translate the distal part to correct for abnormal anteversion angles. **(C)** Patella advancement surgery. It is possible to define the new length of the patella ligament and/or move its insertion on the tibia, during the operation the ligament in the new configuration is shown as a red cylinder.

In an *Extension and Derotation Osteotomy* (Figure 2B), two cutting planes define the bone wedge for removal (Lenhart et al., 2017). In the GUI, the pose of the cutting planes on the desired bone can be defined. The wedge is removed and the remaining bone segments are then reconnected by joining the cutting planes. The intra-segment rotation perpendicular to the cutting planes and the translation along the cutting plane can be specified by the user. Based on bony landmarks, important morphometric information, such as anteversion angle and neck shaft angle of the simulated post-operative bone configuration are visualized in real-time to guide the user in performing the virtual surgery. The *Derotation Osteotomy* surgery is implemented in a manner similar to the *Extension and Derotation Osteotomy* but involves only a single cutting plane on the desired bone. After cutting the bone, the distal part of the bone can be rotated and translated with respect to the proximal part of the bone to correct for bony deformities. Within the *Muscle Transfer* tool (Figure 2A), we provide several options to modify the muscle geometry. These options include adding, removing, translating and changing the muscle attachment, insertion and via points. The resulting change in musculotendon length is translated into a change in tendon slack length. The underlying assumption is that muscle transfer surgeries do not directly affect the muscle fiber architecture, but change the length of the tendon either by removing part of the tendon, lengthening the tendon, or by transferring it to the tendon of another muscle. *Patella Advancement* (Figure 2C) can be performed by either changing the length of the patella ligament, or by transferring the attachment of the ligament on the tibia to a new position. Patella movement is defined as a function of knee flexion angle. After changing the ligament attachment or length, the new path of the patella is determined through an optimization procedure. This optimization procedure defines the rotation and the translations needed to represent the patella movement on the plane perpendicular to the knee joint axis. This optimization finds the patella movement that results in the most constant distance between two points on the patella, one proximal and one distal, and the femur surface throughout the motion while maintaining the patella ligament length constant. *Botulinum Toxin Injections* are modeled by a decrease in the injected muscle's maximum isometric force. This is a highly simplified representation of what botulinum toxin injections do to the muscle and further research is needed to refine this procedure in our model.

Any change applied to a given model results in a new post-operative model, which can be saved for future use.

### Capability Gap and Muscle Report

As previously introduced, the main outcome of the present framework is the capability gap. The capability gap is the difference between the joint moments needed for performing a “desired” motion, i.e., TD walking, and the joint moments the personalized model of the patient (this can be either a pre- or post-operative model) can generate. We use a synergy-based static optimization approach to compute the capability gap. Here, the reserve moments appearing in the moment equilibrium function (Equation 5) represent the torque deficit and hence the capability gap. Subject-specific musculoskeletal geometry and muscle parameters are described in the musculoskeletal model. Impaired motor control is imposed through additional constraints on the activations based on the patient's muscle synergies.

The desired motion used for the computation is derived by scaling average TD walking data to the patient's dimensions (Table 1). First, TD kinematics are imposed to a generic model that was scaled to the patient and corresponding 3D marker trajectories are extracted. The magnitude of the ground reaction forces is scaled based on mass and their point of application, expressed in the foot reference frame, is scaled based on body height. Successively, using the marker trajectories and ground reaction forces, the joint moments required for the personalized model to perform the desired motion are computed by an inverse kinematics and inverse dynamics analysis. In addition, corresponding muscle moment arms and musculotendon lengths are computed. By tracking marker trajectories consistent with TD walking instead of imposing TD joint kinematics directly to the musculoskeletal model, we avoid that the presence of bony deformities leads to unrealistic gait patterns. As an example, if the femoral neck anteversion is 30° higher than normal, imposing TD kinematics to the personalized model would result in a gait pattern with the knee and foot pointing outwards by about 30°, whereas if we track the marker trajectories the knee and foot will point forwards.

Afterwards, the synergy constrained static optimization is performed. The cost function to be minimized is *C*^*SO*^ as defined by Equation (3). The moment equilibrium (Equation 5) has to be satisfied. Inputs to Equation (5), τjiID, rjmi, and l˜mi, are computed based on the patient model and a TD walking pattern as described above. Instead of solving for independent muscle activation patterns, we now solve for synergy activation patterns *H*^*opt*^. The optimization variables are hence Hiopt,τjiR and ^*m*^*W*, which is a deviation from the pre-operative synergy weights (see below). Individual muscle activation patterns (including both subsets *N* and *M*) are then computed from the synergy activation patterns using the synergy weight vectors that describe the patient's motor control *W*^*pre*^ (see section Motor Control):

(14) ami=(Wpre+ΔWm)×Hiopt,

The same muscle co-contraction patterns (*W*^*pre*^) are used to compute the pre- and post-operative CG, since we hypothesize that the orthopedic intervention does not alter muscle co-contraction patterns. Hence, we assume that the neural system will respond to the altered musculoskeletal geometry by changing the timing and magnitude of the pre-operative activation patterns *H*. A small deviation (*W*) from the original weights is allowed because the synergy matrices computed using NNMF typically do not capture 100% of the signal variability. In our formulation, *W* is normalized so that the maximum value in each vector equals one and *W* ≤ 0.05.

Because of the altered musculoskeletal geometry, muscle parameters and synergy-based constraints on muscle activations, it is likely that the muscles cannot generate the TD joint moments. The non-selective motor control imposed by the synergy weights might impose antagonistic co-contractions, hindering the moment generating capacity of a muscle. Alternatively, muscles could be excessively stretched when imposing TD gait kinematics and generate high passive forces. A considerable contribution from the residual actuators might thus be required to satisfy the moment equilibrium. The magnitude of the residuals moments required to match the desired moments defines the capability gap.

**Figure 3:**
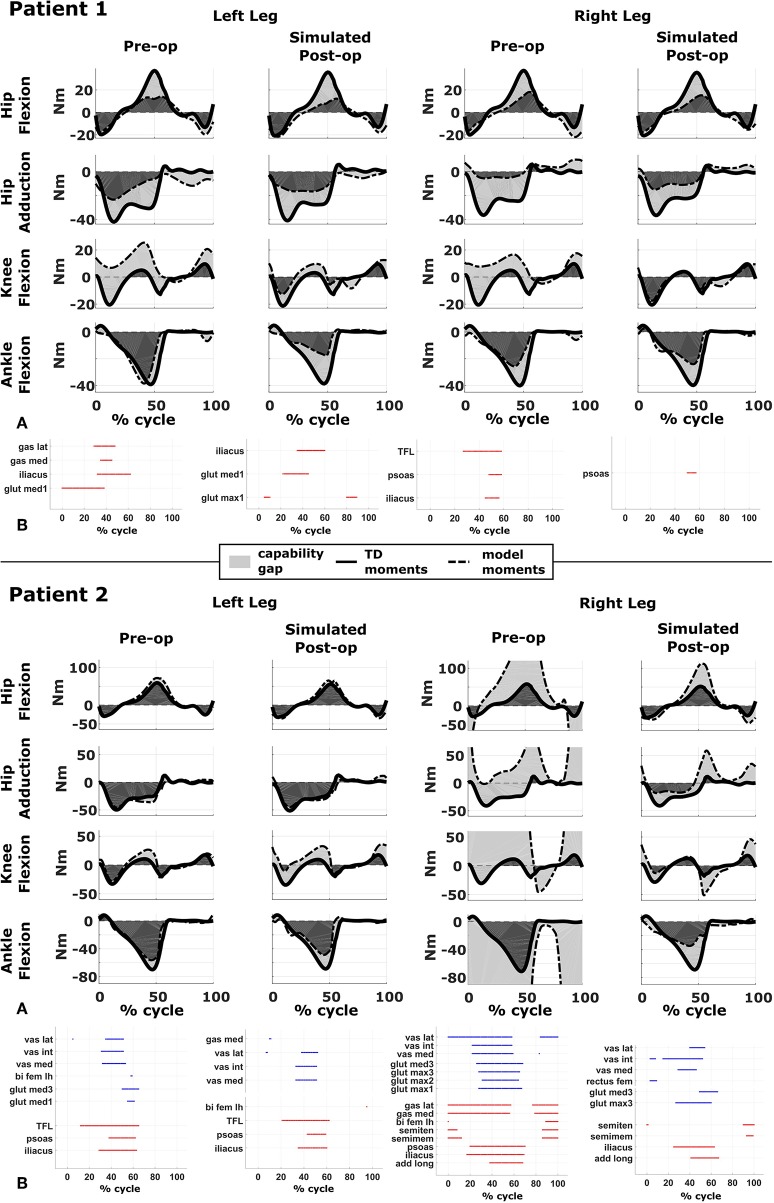
Capability gap and report on muscle operating lengths. **(A)** Capability gap computed for the two representative patients, before and after the virtual surgery. The continuous black lines represent the joint moments required for the model to reach the desired kinematics [i.e., typically developed (TD) gait cycle]. Dotted black lines represent the moment exerted by the model. The light gray patches represent the capability gap. **(B)** Information about the operating conditions of the muscles. Blue dots represent the time instants in which muscles are active at short lengths (activation >0.25 and normalized fiber length smaller than 0.6), red dots the intervals in which muscles are stretched, and exert an excessive passive force (>0.5 times the maximum isometric force).

The CG is represented graphically as a function of the gait cycle for the different degrees of freedom (Figure 3A) and is quantified for each degree of freedom:

(15) CjG=∑i|τjiR|/∑i|τjiID|,

Furthermore, we provide a muscle report (Figure 3B) summarizing the intervals during which muscles operate with excessively short or long fiber lengths. Excessively stretched muscles are those whose passive force is >0.5 times their maximum isometric force (corresponding to $\tilde{l}$ larger than 1.5 *l*^*mo*^). Muscles active at short lengths are those whose activations are >0.25 when operating at normalized fiber lengths smaller than 0.6, meaning that they are producing relatively little force.

### Alternative Analyses

This framework is originally intended to work with the personalized neuro-musculoskeletal models as described above but it is also possible to exclude one or more of the personalization blocks. In fact, this procedure can be of value when assessing the importance of the different factors contributing to the impairment, as highlighted in section Case Studies. It is for instance possible to import a scaled, generic model, and perform the same analyses and surgeries, as envisaged for the personalized models, in order to evaluate the importance of the bony deformities in defining the impairment of the patient. Alternatively, it is possible to investigate alternative causes of the functional impairment by excluding the muscle force/length relationships or the constraints imposed by the motor control on the muscle activations when computing the gait performance (CG).

### Case Studies

We analyzed two representative patients with the proposed framework (Table 1). Both were diagnosed with diplegic CP and underwent SEMLS. For both patients, MRI images were acquired prior to the intervention (for details on the protocol, see Bosmans et al., 2014). A standardized clinical examination protocol (Desloovere et al., 2006) was conducted to evaluate the level of spasticity, strength, selectivity and range of motion. Three-dimensional gait analysis was performed before and after the intervention. Each participant was equipped with a set of reflective markers using the Vicon Plug-in-Gait marker set for lower limbs. Using a 10–15 camera motion capture system (Vicon Motion Systems, Oxford, UK) and two force plates (AMTI, Watertown, MA, USA), marker trajectories and ground reaction forces were collected during one static trial and at least three walking trials at self-selected walking speed. EMG signals were collected (Zerowire, Cometa, Italy) from eight major muscles per leg (rectus femoris, vastus lateralis, biceps femoris long, medial hamstrings, tibialis anterior, gastrocnemius, soleus, and gluteus medius). The local ethical committee approved all procedures, and written informed consent was obtained from the parents of the children prior to participation.

We created pre-operative personalized models using the experimental data (MRI, 3D gait analysis and clinical examination report) and post-operative personalized model by performing virtual surgeries according to the surgical plan of the actual intervention. For Patient 1 the following interventions were modeled: bilateral rectus femoris transfer, distal femur extension, and derotation osteotomy, patella advancement. Patient 1 also received a derotation of the tibia, but this was not modeled due to the fact that the MRI from which the musculoskeletal model was built did not include images of the feet and distal tibiae. For Patient 2 the following interventions were modeled: bilateral rectus femoris transfer, left distal femur derotation, right distal femur extension, and derotation osteotomy, patella advancement, right gastrocnemius and psoas release.

To simulate the *Extension and Derotation Osteotomy* intervention, the angle between the two cutting planes in the femur was modeled based on the knee extension deficit observed when testing the passive range of motion. For the femur *Derotation Osteotomy*, the anteversion angle was corrected to be equal to 0° in the simulated post-operative model in agreement with information provided by the orthopedic surgeons. *Patella advancement* was modeled by shortening the patella ligament by 2 cm, as reported in the surgical plan. *Rectus Femoris Transfer* was modeled in two steps. First, a via point in the femur reference frame was introduced in the middle of the muscle-tendon unit. Second, the insertion site was transferred to the semitendinosus tendon, while keeping the original length of the musculotendon unit unchanged. Our approach replicates the surgical procedure in which the rectus femoris is detached and reattached distally but left attached proximally, thus maintaining its function as a hip flexor. *Muscle Release* interventions were modeled by completely removing the muscle contribution from the generated moment, i.e., by setting the maximum isometric muscle force to zero. Although both patients additionally received botulinum-toxin injections, these were not included in the postoperative model given that their effect can be considered small given the time between the pre- and post-operative observations (10 and 13.5 months).

**Figure 4:**
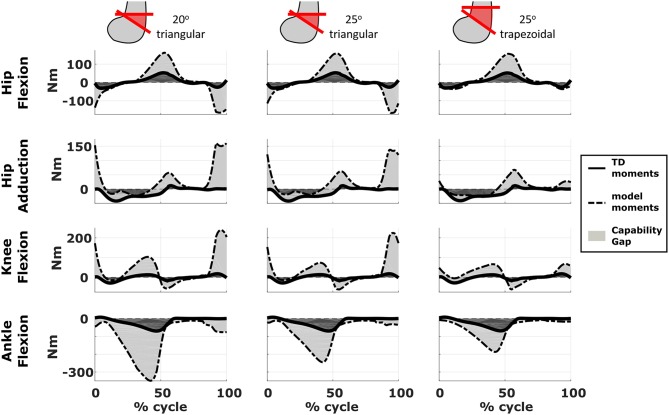
Effect of treatment options on the capability gap. Data reported are relative to the simulated post-operative condition of the right leg of Patient 2. Capability gap is computed after rectus femoris transfer, femur extension and derotation osteotomy, and patella advancement interventions but before performing any muscle release intervention. The continuous black lines represent the joint moments required for the model to reach the desired kinematics [i.e., typically developed (TD) gait cycle].

**Figure 5:**
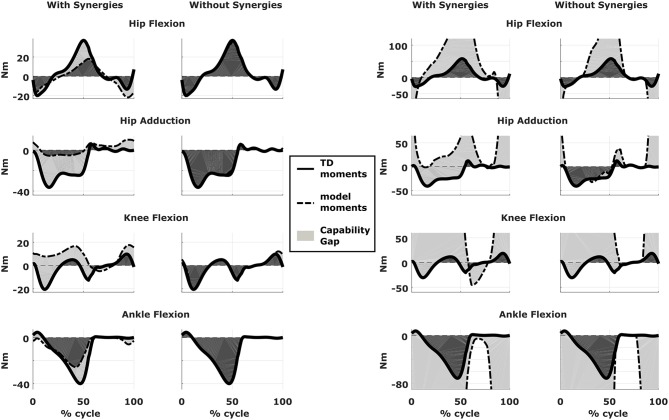
Effect of motor control on capability gap. Capability gap computed relative to the pre-operative condition for the right legs of both patients (on the left side is Patient 1, on the right is Patient 2). The continuous black lines represent the joint moments required for the model to reach the desired kinematics [i.e., typically developed (TD) gait cycle]. When synergies are taken into account the muscle activations are computed from the pre-operative weight vectors, which define the muscle coordination specific to the impairment; when synergies are not taken into account muscles are activated selectively, thus simulating an unimpaired motor control.

**Figure 6:**
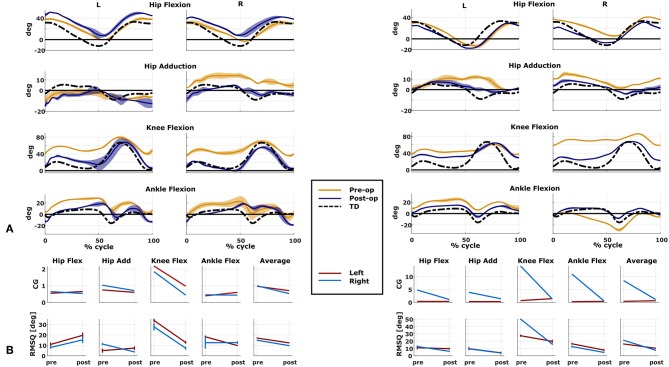
Predicted and measured changes in patient performance. Data on the left belong to Patient 1, on the right to Patient 2. **(A)** Kinematics of the two representative cases measured in both pre- and post-operative conditions (mean and standard deviation). Black dotted line is the average data from typically developed (TD) children used as reference for the computation of the capability gap (CG). **(B)** Changes in the predicted capability gap, and in the root mean square differences (computed with respect to the TD kinematics) between the pre- and post-operative condition.

We compared the predicted motor performances of the patients in terms of the capability gap for the pre-and post-operative conditions (Figure 3). We tested the effect of different treatment options on the capability gap of the right leg of Patient 2 (Figure 4). This was done by creating different models with different angles of the cutting planes defining the extension osteotomy (20° and 25°) as well as two different shapes of the wedge of bone (triangular and trapezoidal). We also investigated the effect of including/excluding the altered muscle parameters and motor control on the predicted gait performance (Figure 5) in the pre-operative condition. When excluding altered motor control from the analysis, muscle activations could vary independently for all muscles. Finally, we tested the ability of our framework to predict post-operative performance by comparing the predicted gait performance (CG) with the gait performance quantified based on the 3D gait analysis performed before and after the intervention (Figure 6). We analyzed the root mean square errors between patient and TD kinematics, corresponding to the Gait Variable Scores and Gait Profile Scores (Baker et al., 2009).

## Results

### Patient 1

The patient had near normal range of motion at the hip and ankle, but a bilateral knee extension deficit in both limbs, bilateral spasticity in most muscles, including rectus femoris, good strength in most muscles bilaterally but slightly lower strength in hip and knee extensors, as well as hip abductors, with overall good selectivity (Table 1). The pre-operative gait analysis (Figure 6A) indicates bilateral excessive knee flexion and ankle dorsiflexion, with incomplete hip extension at the end of stance. The right side hip presents excessive hip adduction.

Synergy analysis revealed that three synergies were sufficient to describe the pre-operative EMG signals in both legs. For comparison, previous work of the group found that, during walking, 57% of TD children use four synergies, whereas the remainder of the subjects uses three. Therefore, to take into account muscles for which no EMG were collected, four synergies were used for the CG computation.

An important CG was found bilaterally at the level of the knees and to a lesser extent hip adduction (Figures 3A, 6B). The simulated interventions were able to reduce the calculated CG, especially at the knees, but also when averaged across all the joints. However, the effect on the CG at the level of the other joints was more variable. In particular, the left hip flexion and ankle dorsiflexion CG showed an increase after the surgery. The post-operative gait analysis shows that knee extension was restored successfully, whereas bilateral hip flexion increased after surgery. Ankle dorsiflexion was restored bilaterally, however, right ankle plantarflexion was still lacking.

**Figure 7:**
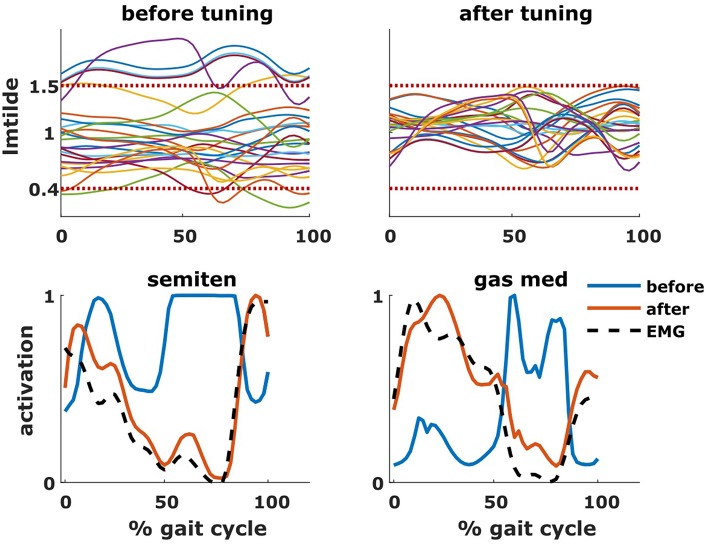
Effect of tuning of muscle parameters. On the top row are plotted the values of the normalized muscle fiber lengths of all the tuned muscles throughout the gait cycle. The two horizontal dotted lines represent the constraints imposed during the optimization process. On the bottom row are plotted the activations of two representative muscles (semitendinosus and gastrocnemius medialis), before and after the optimization, as well as the experimental EMG signal.

The tuning of the muscle parameters was a necessary step to perform the aforementioned analyses. After applying the bony deformities to the model, most of the muscles would have operated at excessive values of $\tilde{l}$ (Figure 7). Therefore, this model would have been unable to generate the required joint moments due to excessive passive forces generation, introducing excessive muscle activations to compensate these and resulting in high residual torques. For Patient 1, residual torques were as high as 45 Nm for the knee joint. Nevertheless, parameter tuning was able to bring the residual torque values below 1 Nm and to produce muscle activations closer to the measured EMG signals (Figure 7).

Within the framework, it is possible to evaluate the isolated effect of motor control deficit, by in- or excluding the motor control model when computing the post-operative CG. In Figure 5, the CG computed in both conditions is presented. For this patient, the CG gap is almost zero when the synergy constraints on muscle activations are not considered. The impaired motor control has thus a major contribution to the CG for this patient.

### Patient 2

The patient's right side was more involved in terms of passive ROM, spasticity and muscle weakness (Table 1). More specific, hip adduction was slightly decreased whereas knee extension and dorsiflexion were more severely restricted in the right limb. Spasticity was present overall, although more pronounced in the distal compared to the proximal muscles. Overall, the patient had good muscle strength, with slightly lower values for the right proximal muscles and hip abductors and for the left hip extensors and plantarflexor muscles. The pre-operative gait analysis revealed bilateral increased knee flexion and hip adduction. Ankle dorsiflexion was increased on the left side, whereas on the right side a reversed second rocker was present. A reversed second rocker is defined by dorsiflexion during loading response and the first half of mid-stance followed by plantarflexion, whereas during normal gait, second rocker is characterized by plantarflexion followed by dorsiflexion. Insufficient hip extension in terminal stance was present at the left hip.

Synergy analysis revealed that four and three synergies could explain the pre-operative EMG signals for the left and right leg, respectively, thus leading to the use of five and four synergies in the CG computations. The CG in the pre-operative condition reflected the reduced range of motion of the right leg. CG was higher for the right then for the left leg with muscles gastrocnemii, hamstrings, iliacus and psoas being excessively stretched (Figure 3B), leading to a large contribution of their passive forces to the CG. In addition, the comparison of the CG computed with and without the inclusion of the motor control (Figure 5) supports the interpretation that the vast majority of the CG is due to the aberrant musculoskeletal geometry and muscle properties, and not motor control.

The simulated treatment had a very different impact on the CG for the two legs. For the right leg, surgery massively reduced the CG. The simulated extension and derotation osteotomy in isolation reduced the CG generated by the hamstrings at the hip and knee at the beginning and end of the gait cycle. The use of a trapezoidal wedge, most commonly used to correct large extension deficits, reduced the CG most by reducing excessive stretch in the muscles (Figure 4). The effect of the extension osteotomy was significant, but even after this treatment a large CG was present. This CG was most elevated in the stance phase for the ankle and knee joint and around toe-off for the hip joint. The muscle report indicated that the gastrocnemii, iliacus and psoas were markedly stretched during these intervals (Figure 3B). These muscles were targeted by the release procedure. The inclusion of these procedures within the framework further reduced the predicted CG as shown in Figure 3A. On the other hand, the pre-operative CG of the left leg was smaller and our framework predicted a slight increase in the CG after the intervention (Figures 3A, 6B).The post-operative gait analysis confirms the positive outcome, with a bilateral marked improvement in the observed kinematics (Figure 6).

## Discussion

We introduced a novel and promising musculoskeletal modeling and simulation-based framework to assist clinicians in the treatment selection process to improve gait function in patients with CP. The salient feature of this framework is a comprehensive personalization of the models comprising subject-specific musculoskeletal geometry and muscle parameters as well as motor control. Furthermore, we introduced a GUI to simulate different orthopedic interventions and interactively modify the musculoskeletal models. As a result, the effect of several candidate orthopedic interventions on the gait performance, evaluated in terms of the patient's capability gap, can be evaluated. In comparison with a number of other studies that aimed to predict the outcome of orthopedic treatments (Hicks et al., 2011; Schwartz et al., 2016; Galarraga et al., 2017), our method differs by the fact that the predictions within our framework are not based on statistical methods. This allows the user to select different treatments or combinations thereof and to evaluate their combined or isolated effects.

In comparison with studies applying forward predictive simulations (Fox et al., 2009; Mansouri et al., 2016), our framework offers the possibility to include a variety of interventions and to fine-tune their parameters: it is for instance possible to combine a muscle transfer or patella advancement surgery with a femoral extension derotation osteotomy and to specify the amount of bony correction. A recently published paper (Lee et al., 2019) proposes a similar framework in which it is possible to predict the post-operative gait after an orthopedic surgery. However, several differences with respect to our work are worth noticing. The model of the motor control used by Lee et al. is obtained via a trajectory mimicking policy, which does not take into account the patient's coordination strategy, whereas we include this feature using EMG based muscle synergies. While in Lee's work subject-specific muscle-tendon parameters are not included, we proposed a tuning based on detailed pre-operative information. In addition, there are differences in the proposed sets of interventions. Both studies implemented derotational osteotomies and muscle transfers, but Lee et al. included muscle-tendon lengthening while we included patella advancement and femoral extension osteotomy surgeries based on the interventions that are commonly used in CP treatment.

We demonstrated the potential of our framework based on two case studies. Indeed, we found a good agreement between the evolution of the predicted motor performance measured with the CG and the actual evolution of the patient kinematics. Using our framework, we were able to highlight the importance of taking into account the specific neurological and musculoskeletal impairments of the patients with CP when assessing gait dysfunction during the planning of an orthopedic intervention.

In Patient 1, virtual simulations of procedures correcting the bony deformities were able to reduce the CG. This agrees with the general trend of improved gait kinematics measured during the post-operative gait analysis. The CG computed without the inclusion of the motor control was almost negligible. Therefore, a hypothetical patient with the same musculoskeletal geometry and properties, but able to activate his/her muscles selectively, could be able to achieve a normal gait pattern. For this patient, the impaired motor control plays thus a major role in determining the altered gait pattern. It is interesting to note that, despite being mainly due to the impaired motor control, the CG of the patient is sensitive to the orthopedic intervention, suggesting an interaction between the motor control and musculoskeletal condition of the patient. In other words, motor control impairments might limit the compensation strategies that are available to patients with CP to counteract musculoskeletal deformities. In contrast, excluding the muscle synergies from the calculation of the CG for Patient 2 had little effect. This indicates that the abnormal muscle parameters, specifically the shortness of several muscles, are the main contributors to the altered gait pattern. This is evident in the muscle report, which indicates that many muscles, including gastrocnemius and psoas, operating at excessive lengths when the CG is high (Figure 3B). These findings support the need for additional muscular interventions on the right side, more specific the gastrocnemius and psoas release. The insights in the two case studies provided by our SimCP framework suggest that the underlying causes for the gait deviations might be very different in different patients, even when they present with similar gait patterns. The constraints imposed by the motor control and the musculoskeletal system should hence be taken into account during the clinical decision process.

Furthermore, we demonstrated that the implementation of a specific orthopedic intervention, more specific the choice of cutting planes and derotation magnitudes, might have a big influence on post-operative gait performance. By creating several models corresponding to different feasible variations in the surgical technique, it is possible to evaluate which variation has the highest potential to reduce the functional impairment of the patient. For example, increasing the angle of the bone wedge reduced the CG in Patient 2, whereas a trapezoid wedge reduced the CG even more. In the future, we plan to develop an optimization based procedure to automatically identify the combination of surgical parameters that minimizes the predicted CG.

The presented framework has still several limitations that will be addressed in future studies. First, there is a need for validation of the model prediction. Although we showed the potential of the framework here, many more cases are needed to demonstrate that the CG is a valid measure of gait performance. To this aim, we will compare the computed change in CG with the measured change in gait kinematics induced by the treatment in a large population of CP children. In addition, the different subcomponents of the framework need further validation. For example, we plan to use MRI-images collected post-operative to validate our implementation of the surgical interventions. Second, all analyses are based on static optimization with musculotendon units having rigid tendons. This approach was chosen to reduce computational time and for ease of implementation. We are currently developing a dynamic optimization implementation that takes into account muscle dynamics enabling the inclusion of a model of muscle spasticity (Falisse et al., 2018). These developments might further improve prediction accuracy. Third, the CG does not predict how the patient will walk in terms of the kinematics. The CG does not describe how a patient will move after treatment but how difficult it would be for him/her to achieve a normal gait pattern. In other words, the CG does not provide any insight in possible kinematic compensation strategies. However, the CG has the advantage of being fast and easy to compute, requiring only a few seconds per trial, and thus enabling the comparison of multiple treatment options. In the near future, we plan to include predictive simulations of gait kinematics in our framework building on the workflow for personalized modeling that we presented here.

A beta version of the developed GUI is freely available (https://simtk.org/projects/simcp), which will enable the biomechanical community to create post-operative models in an easy way and therefore foster future research related to orthopedic interventions and pathological gait. Furthermore, the GUI as well as the concept of the CG can be applied to different populations (e.g., stroke) and research questions (e.g., strength training).

To summarize, we conceptualized and developed a simulation-based framework that relies on highly personalized patient-specific models, including a description of the musculoskeletal geometry, the muscle parameters and the motor control. This framework is designed to assist clinicians in selecting the most promising treatment option for an individual patient solely based on pre-operative data. It is our aspiration that this *in silico*-informed clinical decision making framework will increase the number of positive treatment outcomes in ambulatory children with CP.

## Data Availability

The datasets generated for this study are available on request to the corresponding author.

## Ethics Statement

This study was carried out in accordance with the recommendations of the local ethical committee [Commissie Medische Ethiek KU Leuven (Medical Ethics Committee UZ KU Leuven/Research)]. In accordance with the Declaration of Helsinki, written informed consent was obtained of the participants' parents prior to the experiment. The participants parents supervised the measurement session. The protocol was approved by the Medical Ethics Committee UZ KU Leuven/Research.

## Author Contributions

LP, IJ, FD, KD, and AH contributed conception and design of the study. EP collected the data. AV and GM provided clinical insights for the methods development. LP, HK, AF, MW, SV, and HH contributed in developing the methods. LP, IJ, and FD wrote sections of the manuscript. All authors contributed to manuscript revision, read, and approved the submitted version.

### Conflict of Interest Statement

The authors declare that the research was conducted in the absence of any commercial or financial relationships that could be construed as a potential conflict of interest.
